# Application of three-dimensional printing technology in renal diseases

**DOI:** 10.3389/fmed.2022.1088592

**Published:** 2022-12-02

**Authors:** Shuxin Dai, Qi Wang, Zhiwei Jiang, Chang Liu, Xiangyu Teng, Songbai Yan, Dian Xia, Zhouting Tuo, Liangkuan Bi

**Affiliations:** ^1^Department of Urology, The Second Hospital of Anhui Medical University, Hefei, Anhui, China; ^2^Peking University Shenzhen Hospital, Shenzhen, China

**Keywords:** three-dimensional (3D) printing, kidney, preoperative communication, renal carcinoma, renal calculus, renal transplantation, pharmaceutical research

## Abstract

Three-dimensional (3D) printing technology involves the application of digital models to create 3D objects. It is used in construction and manufacturing and has gradually spread to medical applications, such as implants, drug development, medical devices, prosthetic limbs, and *in vitro* models. The application of 3D printing has great prospects for development in orthopedics, maxillofacial plastic surgery, cardiovascular conditions, liver disease, and other fields. With in-depth research on 3D printing technology and the continuous update of printing materials, this technology also shows broad development prospects in renal medicine. In this paper, the author mainly summarizes the basic theory of 3D printing technology, its research progress, application status, and development prospect in renal diseases.

## Introduction

Three-dimensional (3D) printing technology builds special materials into established models by printing several times based on 3D digital models (1). It originated in 1986 and expanded to the construction and manufacturing industries in the 1990s. In recent years, it has played an increasingly important role in aerospace (2), experimental research (2), medicine and medical treatment (3), and other fields. In the medical field, 3D printing technology is primarily a process of transforming medical images, including computed tomography (CT) (4), magnetic resonance imaging (MRI) (5), and ultrasound (6), into solid models. This is also known as additive manufacturing (AM) or rapid prototyping (RP). It creates 3D objects from stereolithography (STL) files through layer-by-layer continuous superposition using various technologies. In recent years, many countries are attempting to establish a 3D printing industry and apply this technology to various fields, including business, mechanical engineering, and medicine (7). It has great applicability and development prospects in medical fields, such as implant and anatomical models, tissue and organ manufacture, and prosthesis customization (8).

In the last decade, research on 3D printing in medical fields has grown dramatically, covering various aspects such as orthopedics (9, 10), plastic surgery (11), thoracic surgery, cardiac surgery (12, 13), tissue engineering of medical devices, and drug research. Its research and application in urology have also increased significantly and have become a focus of attention and research in the field of urology, especially nephrology (14). Previously, we could only perform preoperative analysis and design of urology surgery through X-ray, intravenous pyelography, CT, and other imaging data, but these imaging data are not as intuitive in reflecting the severity of the lesion site and surrounding tissues and anatomical malformations as they are in the intraoperative eye view. Therefore, surgical success often depends on the surgeon’s exploration of intraoperative lesions and rich clinical practice experience. If the preoperative and intraoperative judgment of the lesion location is inaccurate, it may directly affect the therapeutic effect, safety, and success rate of the operation. At present, 3D printing technology can directly and accurately print the lesion model and anatomical structure of the surgical area according to the preoperative imaging data of patients, such as MRI and CT, and provide patients with customized high-precision surgical plans and implants in the field of surgery. In this way, the success rate of complicated surgical procedures can be improved, more accurate surgical plans can be made, possible intraoperative risks can be assessed in advance, and intraoperative emergency plans can be made. Surgeons can also use it for surgical planning and training in a physical model. Finally, it can make the operation easier and more mature, shorten the operation time, and reduce the risk and failure rates. The maturity and development of 3D printing technology have brought good news to patients with kidney diseases. In view of this, the author reviewed the application of 3D printing technology in renal medicine and summarized its application status and prospects in renal surgery combined with a literature review.

## Technology base

**FIGURE 1:**
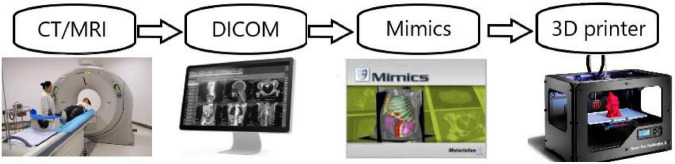
The process of 3D printing technology.

3D printing is a rapid prototyping technology, and a new digital prototyping technology based on computer digital model files. According to the principle of “layered manufacturing, layer-by-layer stacking,” adhesive materials such as powder, metal, and polymer plastic can be accurately printed and stacked layer-by-layer through a 3D printing system to obtain arbitrary complex shaped products (15). Currently, the manufacturing process of 3D printing technology in medicine mainly includes the following steps: (1) The tissue to be printed is examined by CT or MRI, and the data extracted from the imaging files are saved in Digital Imaging and Communications in Medicine format. (2) The sectional image is segmented by Mimics and other software processing systems, and then the printed tissue is extracted by the segmentation method of threshold and region growth. Finally, it is processed by Laplacian smoothing and transmitted to a 3D printer in the STL format. (3) After the 3D printer completes the model printing, it requires 4–6 h to make the model dry and stable. (4) The specific substance completely covers the surface of the model for easy absorption by the model. Model penetration avoids adhesion and poor elasticity between adjacent tissues (4). Compared to two-dimensional (2D) imaging, 3D models can measure anatomical structures more accurately. The resolution of 3D printing technology in horizontal and vertical directions can reach 0.01 and 0.2 mm, which is in line with the resolution of the original data. Therefore, 3D printing technology can meet the requirements of human anatomical structure printing. A basic flowchart of the 3D printing model design is shown in Figure 1.

3D printing technologies can be classified according to the type of technology and material used. Specialized techniques include stereolithography apparatus (SLA), multijet printing, selective laser sintering (SLS), direct metal laser sintering (DMLS), fused deposition modeling (FDM), and so on (3). The material classification includes thermoplastic polymer materials, metals, ceramics, photopolymers, paper, foils, plastic films, titanium alloys, and biological materials (3). In medicine, various kinds of 3D printing can be divided into four types according to their applications: (1) For the preoperative model, only the anatomical structure of the lesion and its adjacent relationship with the surrounding area need to be visually displayed, and the biological properties of the material are not high. SLA and FDM are generally used, such as 3D models of liver cancer (16) and renal carcinoma (17). (2) Personalized internal implants require biocompatible materials and the mechanical properties of the printed structure. SLS and DMLS are used as bone tissue scaffolds (18) and personalized vertebral prostheses (19). (3) Tissue engineering scaffolds require printing materials with good biocompatibility and biodegradability, which can be used for FDM and 3D bioprinting, such as vascular scaffolds (20). (4) For cellular structures or tissue-like organs, scaffolds and cells must be printed at the same time, and the operating environment has high requirements; therefore, 3D bioprinting is adopted, such as cartilage tissue-like structures (21) and tissue units (22).

## Preoperative communication and education

**FIGURE 2:**
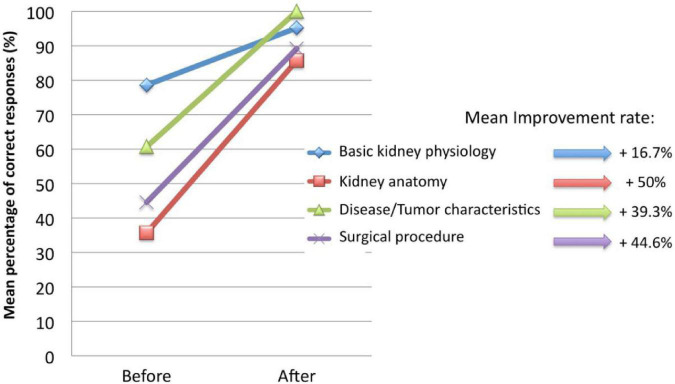
Patients’ understanding changes in several aspects before and after using the 3D printing model (23).

In recent years, more 3D printed models have been used for preoperative doctor-patient communication and education, as shown in Figure 2, helping patients and their families to understand the condition and surgery from many aspects and achieve better doctor-patient communication. For example, the University of Southern California urology Institute has completed a doctor-patient communication study on a 3D-printed renal carcinoma model; their research found that the communication effect of the 3D-printed renal carcinoma model was significantly better than a simple text explanation (23). Similarly, Zhang et al. (24) also conducted a doctor-patient communication evaluation study using a 3D-printed renal carcinoma model, proving that the model is conducive to understanding the diagnosis and treatment process by patients and their families. The 3D model recreated the entire lesion organ and its adjacent structures. It facilitates doctor-patient communication and enables patients and their families to understand the entire treatment process. More importantly, it is conducive to active cooperation in the treatment and follow-up care after discharge.

Clinical medicine has strong practicality and applications. The perfection of the teaching system directly affects the cultivation of high-quality talents. Traditional clinical teaching methods are relatively simple, abstract, and lack visualization. Although some multimedia teaching can meet students’ visual learning needs, it cannot achieve hands-on practice, and it is impossible to have enough patients for students to learn and operate clinically. Therefore, many young physicians lack the tactile and 3D anatomical experience that surgery can provide. 3D printing model perfectly compensates for this defect. It can display 2D images in the form of objective 3D objects and provide 3D tactile experience and simulate surgical operations, which is of great positive significance for the technical maturity of young physicians and medical students. Compared with 2D scans, the benefits of 3D-printed models for educating patients, medical students, and physicians have been initially demonstrated. Knoedler et al. (25) asked first-year medical students to complete the RENAL nephrometry score according to CT and 3D models and compared the results. The experimental results showed that the 3D model significantly improved the trainees’ renal function scoring accuracy. It showed consistency among trainees and less difference than expert surgeons, indicating that the 3D model enhanced students’ understanding and familiarity with kidney diseases. The 3D printing technology can be individualized for different cases, and the model can clearly reflect the lesion site and adjacent anatomical relationships. More importantly, it can simulate a real surgical field completely. Using this individualized model in teaching can make students more intuitive in understanding the disease and more skilled in related operations, thereby greatly improving the quality of teaching. Of course, 3D printing also has some limitations in clinical communication and teaching. This new approach is an unprecedented challenge for physicians, patients, and medical students. It not only requires doctors to conduct more in-depth analysis and explanation based on the actual model, but also requires medical students and patients to adapt and cooperate with this new method. We believe that good interactive communication and a certain ability to understand 3D space are the keys to the success of 3D printing in communication and education.

## Medical equipment and surgical implants

**FIGURE 3:**
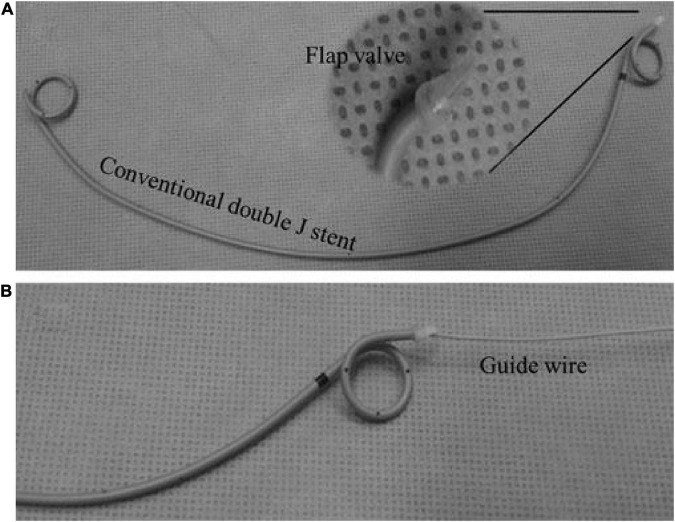
Panel **(A)** shows an anti-reflux ureteral stent, and panel **(B)** shows it attached to a guide wire (28).

The development of 3D-printed medical devices in urology is not as advanced as that in orthopedics, and it is still in the research and development stage; however, some progress has also been made in recent years. George et al. (26) used 3D printing to manufacture hemostatic forceps, needle drivers, scalpel handles, retractors, and forceps and asked urologists to conduct simulated surgical evaluations and constantly adjust the design and improve the method. Del et al. (27) used 3D printing technology to produce personalized ureteral stents and performed perfusion experiments with traditional standard stents in an isolated pig urinary system. They then performed statistical analyses of the total, extraluminal, and intraluminal flows. In conclusion, the effect of 3D-printed ureteral stents was comparable to that of standard stents. We believe that with the continuous upgrading of technology, the application of ureteral stents tailored to patient anatomical characteristics is not far away in clinical practice. Park et al. (28) reported a more active mindset. Based on Del et al.’s report, they used 3D printing to produce a new type of ureteral stent with a polymeric flap to protect against reflux, as shown in Figure 3. The results of the *in vitro* experiments showed that this new type of stent effectively prevented the reverse flow of urine and minimized the reduction in forward flow. The technology of 3D printing surgical instruments and implants is not yet mature, and there are difficulties, such as material selection, material mixing ratio, and printing time control. To date, this technology needs to be explored and researched. In addition, whether it is medical equipment or implants, most current studies focus on effectiveness and lack safety evaluation (27). We believe that as more human and animal experiments are carried out, the practicability and safety of 3D printing in this aspect will be further confirmed.

## Renal carcinoma

**FIGURE 4:**
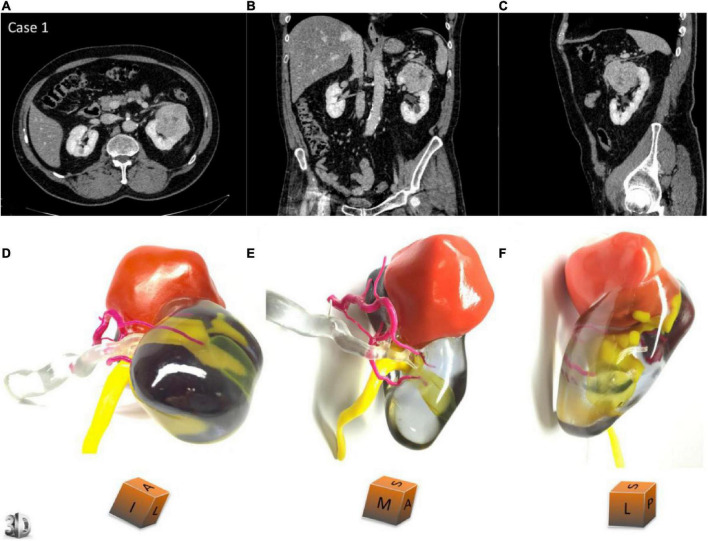
Panels **(A–C)** show the transverse section, coronal plane and sagittal plane of the patient’s kidney during the development phase of CT. Panels **(D–F)** are the corresponding 3D printing models, respectively (23).

A clear understanding of renal tumors and their relationship to neighboring structures is critical to developing surgical strategies and outcomes, and the 3D printing of renal carcinoma models can well help physicians to achieve this goal (25). A specific case is illustrated in Figure 4. As shown in the figure, the 3D model can comprehensively show the location, size, relationship with the collecting system, and distribution of the blood vessels of the tumor. These models, combined with CT and other related imaging data, help the surgeon make the best decision on the surgical approach and accurately resect the lesion and preserve the nephron as much as possible. With technological advancement, 3D printing will provide sufficient technical support for partial nephrectomy in the future, reduce technical difficulties, and promote this surgical method, so that more patients can receive effective treatment.

There are many applications of 3D printing technology in renal carcinomas. Komai et al. (29) successfully performed a minimally invasive partial nephrectomy using a 3D-printed kidney model. The results indicated that the tumor and margin of the surgically resected specimens were almost identical to those of the 3D printed model, demonstrating the feasibility of 3D printing technology for preoperative planning and intraoperative navigation of partial nephrectomy. Wake et al. (5) studied the influence of 3D printing model on preoperative planning of surgery for the complex renal carcinoma. The outcomes proved that compared with the 2D image data, the operation plan made by the surgeon according to the 3D model had a high degree of matching with the actual operation. The 3D model helped us refine the preoperative plan and preserve as many nephrons as possible during the operation. Bernhard et al. (23) printed seven renal carcinoma models for preoperative planning and treatment notification. Through their research, the patients’ cognition was improved, and it also provided some tips and help for doctors to perform renal carcinoma surgery to a certain extent. Many studies have shown that the 3D kidney model is helpful in accurately locating tumors and reducing surgical risks and complications, which is worthy of clinical application. On the other hand, we also see some shortcomings in the application of 3D printing in renal carcinoma. Considering the physiological complexity of tumor tissue, the printed model of kidney cancer lacks the texture and hemodynamic characteristics of the natural organ, which may not fully reflect tumor details. This can lead to some errors and deviations in the preoperative plan. With the development of our understanding of cancer and kidney, it is reasonable to believe that these limitations will be optimized and avoided in the near future.

## Renal calculus

Percutaneous nephrolithotomy is an important treatment method for complex renal calculi. The design and establishment of the percutaneous nephrolithotomy puncture channel are key to the treatment outcomes. We usually judge the general situation of calculi according to preoperative CT and intraoperative B-ultrasound and then establish the channel in combination with clinical experience. Usually, because surgeons do not intuitively and accurately understand the anatomical relationship between perirenal organs, renal vascular distribution, and stone location, it is difficult for them to find the optimal puncture channel, leading to a high incidence of complications such as bleeding, adjacent organ injury, and urinary sepsis (30). However, with the application of 3D printing technology in renal calculus diseases, preoperative stone models can help us understand the shape of calyces and the details of calculi more comprehensively. This is helpful for doctors to choose the best puncture channel before surgery and to improve the accuracy of puncture to reduce surgery-related complications.

**FIGURE 5:**
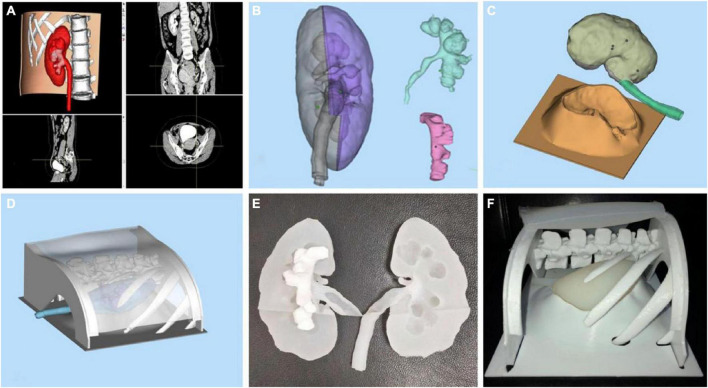
The 3D-printed model of kidney stones. Panel **(A)** is the software modeling. Panels **(B–D)** are the model constructions before printing. Panels **(E,F)** are 3D printed models of kidney related structures and stones (33).

Relevant studies have demonstrated the importance of 3D printing in treating renal calculi. Kang et al. (31) reported that the specific location of renal calculi could be observed using 3D printing technology. The surgeons planned and simulated the operation on the 3D printed model to make the operation more accurate. They could avoid blood vessels, quickly locate the stone, and treat it accurately. According to one study, 3D printing technology has significantly improved the success rate of surgery by avoiding unnecessary surgical procedures and significantly reducing the operation time. Orecchia et al. (32) developed a series of 3D printed models of upper urinary tract and stones to train retrograde intrarenal surgery for renal calculus disease. The models they designed have the advantages of anatomical similarity, low cost and repeatability. What’s more, it can improve surgical skills in a risk-free environment. This reduces the number of cases required for doctors to achieve surgical proficiency while ensuring patient safety to some extent. Xu et al. (33) printed three models for these 12 patients included in the trial and selected three puncture points from the kidney’s upper, middle, and lower calyx to simulate percutaneous nephrolithotomy. They recorded the stone clearance rate and used the puncture site that yielded the highest stone clearance rate in the actual surgery. The results showed a high consistency and correlation between the postoperative calculus volume of patients and the models. It can be seen that a 3D printing model and *in vitro* simulation technology can help us select calyces and establish the best renal puncture channel in the treatment of complex calculi. Successful cases using 3D printing technology in the field of renal calculi are shown in Figure 5. The printed model provides more favorable information for preoperative planning, surgical simulation, and preoperative stone location, as well as support for selecting the best puncture point and angle to guarantee surgical success. While there are many advantages, we should also see some limitations of 3D printing in the application of renal calculus. The position of renal calculus in the human body is not static even in a short time, so the specific situation of the stone shown by 3D printing may not be exactly the same as what is seen during surgery. Scheduling surgery as soon as possible may be one way to solve this problem. In addition, it is also a problem to consider whether different parts and different components of stones need to be represented by different printing materials. All in all, 3D printing technology undoubtedly plays an important role in the diagnosis and treatment of renal calculus, and its development prospects are quite broad as well.

## Renal transplantation

Surgeons have difficulty performing vascular anastomosis during renal transplantation in patients with renal failure due to renal disease and other risk factors. Preoperative imaging also makes it very difficult to evaluate numerous vascular calcification foci, posing great obstacles to renal transplantation. The current international supply of renal transplants is far from meeting demand, and with the development of 3D printing in renal diseases, we can save several lives without the need for donors. We believe this will completely free patients with chronic renal insufficiency from dependence on organ donors and hope for renal transplantation.

**FIGURE 6:**
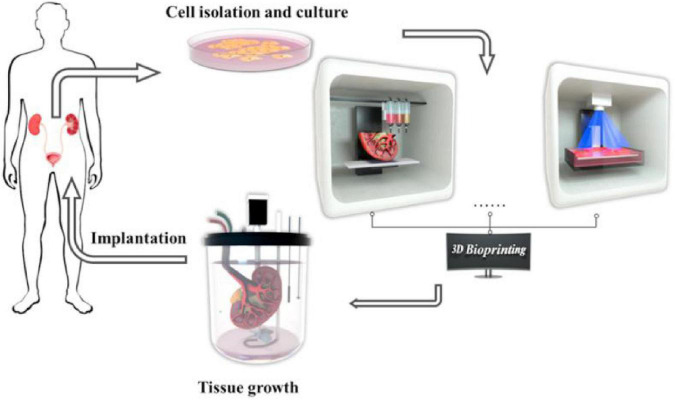
Tissue engineering and three-dimensional (3D) bioprinting technologies for urological diseases (36).

At present, the research of 3D printing in renal transplantation is well underway. Denizet et al. (34) reported that 3D printing technology can reduce the operation time and the incidence of complications at the anastomotic site, and provide help for surgeons in renal transplantation. Chandak et al. (35) pointed out in their study that for children with end-stage renal disease, it is difficult to transplant adult-sized living donor kidneys into children. Three children with unique complex surgical anatomy received living kidney transplants using 3D-printed models. In this experiment, the 3D printing model enhanced the preoperative review and surgical simulation, and all the surgeries were successfully completed. At the most recent follow-up (>16 months), all patients had good renal transplant function. With the aggravation of the aging of society, the number of patients with chronic renal insufficiency is increasing day by day, and the demand for renal transplantation is increasing naturally. The number of organ donations has remained stable or decreased slightly, resulting in a shortage of kidneys. As shown in Figure 6, 3D printing offers hope for kidney transplants by printing functional tissues and even organs (36). However, the kidney generated by 3D printing is still faced with some problems and limitations in the real application of renal transplantation, such as whether the materials used will cause rejection, whether the printed kidney structure is highly compatible with the body. This requires not only a breakthrough in the development of biological materials, but also a deep understanding of the renal anatomy and microenvironment. The 3D-printed tissues and organs have not yet been used in the clinic, but we believe that with the development of related technology, we will be able to reap the fruits of this technological progress.

## 3D bio-printing

With the development of technology and the update of materials, the combination of various biological components and 3D printing technology produces an emerging technology, called 3D bio-printing technology (37, 38). It successfully processes cell-filled bio-inks into tissues and organs with the advantages of automation, high precision, printing adaptability, reproducibility and repeatability on a wide range of materials (37, 38). We believe that it will have broad applications in disease modeling (39), organ-on-a-chip (40), drug discovery and testing (41), high-throughput screening (42), and regenerative medicine (43).

**FIGURE 7:**
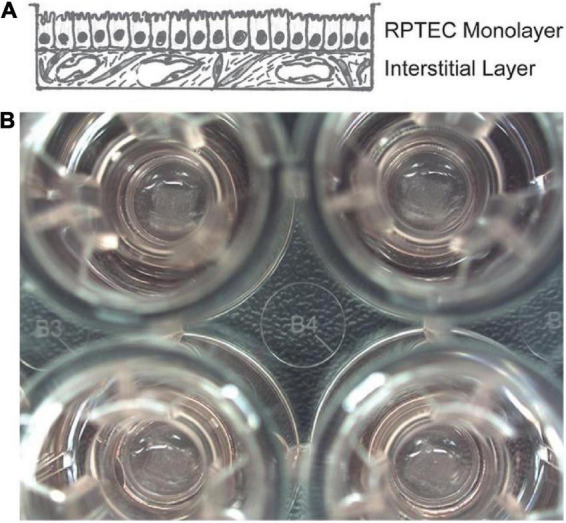
The 3D model of the interstitial interface of the kidney tubule using 3D bio-printing. Panel **(A)** is a hand-drawn view of the supporting epithelial and multicellular interstitial layers. Panel **(B)** is a macro view of the 3D bio-printed proximal tubule tissue in the Transwell experiment on a 24-well plate (41).

3D bio-printing technology has a wide range of applications in the construction of functional tissues, including multi-layer skin (44), bone (45), vascular grafts (46), trachea (47), myocardial tissue (48), and cartilage structures (49). They can replace damaged or diseased tissues to meet the need for suitable tissues and organs for transplantation. Organ transplantation is currently the best treatment for ESRD; however, the number of existing kidney supplies is far from meeting the increasing demands of the ESRD population. Organovo created the world’s first whole-cell kidney tissue using 3D bio-printing. King et al. (41) used 3D bio-printing technology to create a 3D proximal tubule tissue model *in vitro*, as shown in Figure 7. Their research has undoubtedly opened the door to kidney regeneration and organ transplantation. 3D bio-printing of kidney organs is a new technology aimed at developing and printing organs with kidney function (50). In the near future, the emerging technology is expected to be combined with specific biological materials and tissue regeneration engineering to print kidney organs with normal physiological function, thereby improving the current situation of kidney organ shortage and the quality of life.

## Discussion

Some important research and application progress of 3D printing technology in nephrology are summarized in Table 1. The application of 3D printing technology in renal diseases is still limited. Relevant research and applications have not been conducted in the human body on a large scale, and the development prospects are quite broad.

**TABLE 1:** Some research progress of 3D printing technology in renal diseases.

References	Study purpose	Sample size	Imaging modality	Technology/ software	3D printer	Types of printing	Materials	Key research points	Application scope in renal disease
Bernhard et al. (23)	To investigate the application of 3D printing model in self-cognition and doctor-patient education of renal carcinoma patients.	7	CT	Image recognition algorithm Synapse 3D.	Object 500 Connex 3 3D printer.	Stereolithography apparatus (SLA)	Photopolymer material	3D printing technology helps patients learn about their renal carcinomas and surgery before surgery, helps patients educate and improve their satisfaction.	Renal carcinoma
Monda et al. (51)	To explore the effectiveness of 3D printed silicone renal model as an educational resource for physicians.	24	MRI	The open source segmentation software, Invesalius.	Object Eden260VS printer.	Fused deposition modeling (FDM)	Silicone material	The use of 3D printed silicone renal carcinoma model is beneficial to preoperative simulation and improve the technical skills of trainees.	Renal carcinoma
Lee et al. (52)	To assess the application of 3D printing technology in partial nephrectomy and medical student education.	20	CT	Compact View III Ver. 1.03. Optimum Solution, Korea, Blender v2.76. Blender foundatio, Amsterdam, NL.	Object 260 Connex 3.	Stereolithography apparatus (SLA)	Photopolymer material	Personalized 3D kidney models have significantly improved our knowledge of proper kidney anatomy and can be used to develop perioperative and educational programs.	Renal carcinoma
Wake et al. (5)	To determine the influence of 3D printing model on preoperative planning of complex renal mass surgery.	10	MRI	Mimics 16.0	Stereolithography technology using Connex 500 3D printer.	Stereolithography apparatus (SLA)	Flexible, transparent material	The physical 3D model of preoperative MRI may be helpful for the surgical planning of complex renal carcinoma.	Renal carcinoma
Komai et al. (29)	To report the specific 3D surgical navigation in minimally invasive off-clamp partial Nephrectomy (PN).	10	CT	Computer-aided design (CAD) software	Multi-material 3D printer (Objet Connex500, Stratasys Ltd, MN, USA).	Biotexture modeling technology	Acrylic resin material	The use of this specific 3D model can help doctors perform PN surgery and patients understand their surgery.	Renal carcinoma
Park et al. (28)	To describe the design of an anti- reflux ureteral stent with a polymeric flap valve and the fabrication methods using 3D printing.	1	CT	SolidWorks software	Objet500 Connex printer; Stratasys Ltd. Minneapolis, MN.	Selective laser sintering (SLS)	Polyethylene material	The 3D printed stent effectively prevented backward flow while minimizing reduction in forward flow.	Ureteral stents for renal calculus
Orecchia et al. (32)	To study the feasibility of 3D printing of urinary tract and calculi.	6	CT	Mimics 23.0	A A2v4 3D printer	stereolithography (STL)	Water soluble polyvinyl alcohol	3D printed upper urinary tract and stone models are suitable for training retrograde intrarenal surgery for renal calculus.	Renal calculus
Xu et al. (33)	To explore the feasibility and effectiveness of using 3D printing model in surgical planning for total staghorn calculi.	12	CT	Kimics-3D 1.0	Multi-material connex 3D printer.	Fused deposition modeling (FDM)	Gypsum and silicone	3D printing model is helpful for preoperative planning of total staghorn calculi.	Renal calculus
Denize et al. (34)	To study the application of 3D printing in the mouth of vascular anastomosis for renal transplantation.	4	CT	3D-slicer software	multi-jet printer	stereolithography (STL)	Translucent soft resin	3D printing can reduce the operation time and complications of renal transplant anastomosis.	Renal transplantation
Chandak et al. (35)	To investigate the application of a new patient-specific 3D printing in complex living donor pediatric renal transplantation.	3	CT and MRI	Mimics Medical v18.0 software; CAD 3-Matic Medical v.10.0.	Objet500 Connex 1 3D printer.	PolyJet printing	Acrylic polymer material	Patient-specific 3D printing plays an important role in enhancing preoperative planning for pediatric living donor renal transplantation.	Renal transplantation

Although the application time of medical 3D printing technology in drug development and nephrotoxic drug screening is relatively short, its technological advantages and application potential are likely to play a significant role in promoting its development in these fields. 3D printing technology is widely used in the development of drug delivery systems (53), such as controlled-release (54) and sustained-release (55) drugs. Its application is to change the drug release mode and dosage form to apply the minimum drug dose to achieve an effective blood concentration and reduce drugs’ systemic toxic side effects. Cyclosporine A (CsA) is an immunosuppressive agent. Song et al. (56) successfully printed a drug carrier loaded with a CsA polymeric microsphere gel and equipped it with a 3D-printed anti-stress framework, effectively inhibiting animal immune rejection for 4 weeks. It can achieve the purpose of local, continuous, and effective medication, effectively treating the primary disease and reducing the drug dosage and potential adverse effects. This study is currently limited to animal experiments, and the selection of drug excipients in the printing process is limited (57). A series of issues, such as effectively controlling drug release and drug release time, require further study. The pathogenesis of glomerular-related diseases is closely associated with abnormal immune responses. Currently, glucocorticoids and immunosuppressants are the mainstay drugs; however, they have many side effects. Therefore, it is very important to reduce drug dosage and blood concentration. 3D printing technology still has many limitations and deficiencies in drug research. First of all, 3D printing technology for individualized manufacturing according to patients is not very consistent with the need for mass production of drugs (53). Secondly, drugs based on 3D-printed kidney models are undoubtedly more expensive, which also limits their development. We believe that with the optimization of material selection and the progress of printing technology, these problems can be solved to some extent. Although there are many problems to be solved in the application of 3D print-loaded multi-polymer drugs for the treatment of renal diseases, 3D printing technology is still expected to bring about a revolution in drug applications in the direction of renal drug development.

Traditional kidney microarray (58) is a technology used to study the effect of drugs on renal cell function *in vitro* using a 2D model with a single-layer microfluidic system of renal proximal tubular epithelial cells. This technique can only study the effects of drugs on renal proximal tubular cells. The research system lacks the physiological characteristics of a normal renal tissue 3D structure, which cannot fully reflect the physiological microenvironment of the human body and lacks objectivity for drug screening and toxicity tests (59, 60). Early 3D *in vitro* perfusion models (61) required highly specialized manufacturing techniques, which limited their routine application. The combination of renal cell chip technology and submillimeter 3D printing technology extends the structure of the renal function chip system to three dimensions. This can make it closer to normal human physiological kidney function, which is more conducive to the accurate and timely detection of drugs with deleterious effects on the kidney. Homan et al. (62) reproduced a microenvironment similar to the *in vivo* phenotype and function of human proximal renal tubular cells through 3D bioprinting and 3D cell culture combined with organ chip manufacturing technology. This type of kidney chip made by 3D technology can be applied to the drug preclinical trial stage, improving the screening rate and prediction ability of drugs with harmful potentials to the kidneys and avoiding animal experiments. Moreover, it can significantly boost new drug development by reducing the cost of developing new, safer drugs. Although research has only been done on a chip, it has opened up a new avenue for biological 3D printing of kidney tissue structures.

In conclusion, 3D printing technology has gradually gained attention for treating renal diseases. It has shown great value in medical staff training, patient education, surgical planning, and preoperative simulation. However, development prospects in drug research and transplantation are also exciting. Of course, there are some limitations to this technique, especially for patients with chronic kidney disease or allergies to contrast media that make imaging modeling difficult. In addition, when we do 3D print modeling using the traditional methods of native CT scanning, manually delineating the area of interest is often time consuming and not always reasonable. Solutions to these drawbacks are also prospects for future development. Although 3D printing has many shortcomings, such as material, cost, lack of professional personnel, limitations of imaging technology, and patient factors, it will continue to be improved with the development of science and technology, and it is expected to achieve comprehensive individualized treatment of 3D models. In addition, 3D bioprinting using tissue cells as materials will be an epoch-making innovation in the field of renal medicine. We believe that printing cells, tissues, and organs and successfully transplanting them into the human body will contribute to the development of medical science from the perspective of precision medicine.

## Author contributions

LB proposed the idea and general direction of this research. SD wrote the manuscript with the advice and help of other authors. All authors contributed to the article and approved the submitted version.
